# The Impact of COVID-19 Infection on Patients with Chronic Diseases Admitted to ICU: a Cohort Retrospective Study

**DOI:** 10.1007/s44197-023-00112-5

**Published:** 2023-05-18

**Authors:** Farah Alammari, Batla S. Al-Sowayan, Bayan Albdah, Arwa A. Alsubait

**Affiliations:** 1 Department of Blood and Cancer Research, King Abdullah International Medical Research Center, Riyadh, Saudi Arabia; 2 Section of Biostatistics, King Abdullah International Medical Research Center, Riyadh, Saudi Arabia; 3 Medical Research Core Facility and Platforms Department, King Abdullah International Medical Research Center, Riyadh, Saudi Arabia; 4 Clinical Laboratory Sciences Department, College of Applied Medical Sciences, King Saud Bin Abdulaziz University for Health Sciences, Riyadh, Saudi Arabia; 5 King Saud Bin Abdulaziz University for Health Sciences, Riyadh, Saudi Arabia

**Keywords:** COVID-19, CVD, CKD, Cancer, ICU

## Abstract

The coronavirus disease (COVID-19) infection is causing significant morbidity and mortality rates worldwide. A comprehensive investigation of the disease characteristics, especially among vulnerable disease groups, could help better manage the disease and reduce the pathogen's effect. This retrospective study examined the impact of COVID-19 infection on three groups of patients with chronic diseases. We investigated the clinical characteristics and outcomes of 535 COVID-19 patients with cardiovascular diseases (CVD), chronic kidney diseases (CKD), and Cancer that were admitted to the Intensive Care Unit (ICU). Of the total cases, 433 patients (80.93%) were discharged from the ICU, and 102 patients (19.06%) were declared dead. Patients’ symptoms, their clinical laboratory findings, number and type of medications, length of ICU stay, and outcome were collected and analyzed. Most COVID-19 patients included in our study were associated with other comorbidities such as diabetes mellitus, hypertension, and heart disease and failure. Upon ICU admission, the main COVID-19-related symptoms in CVD, CKD, and cancer patients were cough (55.73, 50.42, and 50.5%, respectively), Shortness of Breath (SOB) (59.38, 43.1, and 43.7%, respectively), and fever (41.15%, 48.75%, and 28.2%, respectively). In terms of lab findings, D-dimer, LDH, and inflammatory markers, in particular, were outside the normal range. Treatment options for patients with COVID-19 in ICU were mainly antibiotics, synthetic glucocorticoids, and Low Molecular Weight Heparin (LMWH). Furthermore, CKD patients had a longer ICU stay (13.93 ± 15.87 days) which illustrates the poorer outcome in this group of patients compared with the others. In conclusion, our results highlighted the significant risk factors among COVID-19 patients within the three groups. This can guide physicians in prioritizing ICU admission and help in the management of critically ill patients with COVID-19.

## Introduction

The coronavirus disease (COVID-19), caused by severe acute respiratory syndrome coronavirus 2 (SARS-CoV-2) [1], has been a universal hot topic since early 2020. In January, the World Health Organization (WHO) general director declared the COVID-19 outbreak a public health emergency of international concern [2]. Like prior coronavirus outbreaks, Severe Acute Respiratory Syndrome (SARS, 2002) and the Middle East Respiratory Syndrome Coronavirus (MERS-CoV, 2012), COVID-19 is mainly a respiratory tract infection and spread via droplets and tiny airborne particles. However, by March 2020, the COVID-19 outbreak was characterized as a pandemic due to its alarming spread and severity [3]. Shortly after, in April 2020, 1 million global cases of COVID-19 were confirmed. Then by the end of September of the same year, the death toll passed the 1 million mark [4]. COVID-19 symptoms manifest two to fourteen days following virus exposure, including a clinical spectrum ranging from asymptomatic, mild, to severe symptoms. The most common symptoms are fever, cough, fatigue, headache, and loss of taste or smell. However, severe symptoms include shortness of breath and chest pain [5]. The case fatality rate (CFR) for COVID-19 ranges vastly between different countries. For example, in November 2021, CFR ranged from 0.1% to 19% across different world areas[6]. Several factors contribute to the CFR discrepancy independent of the disease itself. These include political and economic, in addition to demographical factors. However, despite their geographical location, individuals with chronic health conditions tend to be more vulnerable to the virus, develop more severe complications, and are more likely to die due to the COVID-19 infection. This study examined the infection in three major high-risk groups: people with cardiovascular diseases, chronic kidney diseases, and cancer.

Cardiovascular diseases (CVDs) are pathologies that affect the heart muscle or the blood vessels. According to the WHO, CVDs are the leading cause of death worldwide. It is estimated that 32% of global deaths are due to a form of CVDs [7]. During the pandemic, the routine and emergency health care provided to CVDs patients was severely interrupted due to the increasing number of COVID-19 patients that burden healthcare systems worldwide. The paralyzed healthcare system alone has made people with CVDs more vulnerable than people with no underlying medical conditions. It has been shown that the SARS-CoV-2 virus causes cardiovascular-related complications through different mechanisms. The virus could directly infect the cardiac muscle through blood circulation. In addition, SARS-CoV-2 receptor angiotensin-converting enzyme 2 (ACE2) is found abundantly in the lung and the heart, which serve as an entry gate for the virus [8]. COVID-19 was also reported to indirectly affect the cardiovascular system through increased production of pro-inflammatory cytokines [9]. For people who already have CVDs and contract SARS-CoV-2, the risk to their already compromised cardiovascular system becomes higher. It was reported that COVID-19 patients with CVDs are more susceptible to experiencing more severe symptoms. For example, a meta-analysis study (six studies with 1527 patients) reported that 17.1% and 16.4% of COVID-19 patients had hypertension and cardia-cerebrovascular diseases. In addition, COVID-19 patients with these conditions are two to three folds more susceptible to intensive care unit (ICU) admission [10]. COVID-19 patients with CVDs are also expected to have a poorer prognosis. The Chinese Center for Disease Control and Prevention reported that the overall CFR of the 44,672 confirmed SARS-CoV-2 cases was 2.3%. However, the CFR jumped to 10.5% in patients with CVDs compared to CFR of 7.3% in patients with diabetes, 6.3% in patients with chronic respiratory disease, and 5.6% in patients with cancer [11].

The second group examined in this study is people with chronic kidney diseases (CKDs). CKDs are defined as decreased kidney function accompanied by a progressive decrease in glomerular filtration rate (GFR) [12]. CKDs can range from mild to severe chronic kidney failure. It was estimated that the global prevalence of CKDs in 2017 was 9.1%, which is a 29.3% increase compared to 1990 [13]. Several factors could cause CKDs; however, CKDs are closely associated with diabetes mellitus, hypertension, obesity, and aging [14], which explains the global increase in CKDs cases. Acute kidney injury (AKI) is common among COVID-19 patients in general, with the risk of developing AKI being closely related to the severity of the infection [15]. The AKI could occur directly via SARS-CoV-2 entry through its receptor ACE2, which is present in the tissues of the kidney. Alternatively, COVID-19-induced AKI could occur due to indirect mechanisms like heart failure, nosocomial sepsis, or antiviral drug use [16]. For people who already have CKDs, the situation becomes even direr. In addition to facing an interruption to their CKD-related medical care, it was reported that people with CKD are more susceptible to contracting COVID-19 and are more likely to develop a severe form of infection [17]. Moreover, some sub-groups of CKD patients are at higher risk of contracting the infection than others. These sub-groups include patients on hemodialysis and those who received a kidney transplant and are on immunosuppressants. Furthermore, when contracting COVID-19, patients with CKDs have a significantly higher mortality rate compared with COVID-19 patients with no underlying renal condition [18].

The third high-risk group investigated in this study is people diagnosed with cancer. Cancer is the second leading cause of death worldwide, with the most cancer-related death resulting from lung, colon/rectum, and liver cancers [19]. Regardless of cancer type and prognosis, people with a cancer diagnosis are predicted to have poorer outcomes when infected with SARS-CoV-2 [20]. For a start, similar to other people with chronic conditions, cancer patients experienced delayed access to their cancer-related medical care during the pandemic [21]. The retarded treatment process was detrimental to this sub-group's physical and mental well-being [22]. In addition, cancer patients are more vulnerable to infections in general due to their immunosuppressed status. The compromised immune system in cancer patients is usual and could result from a neoplasm or anti-cancer treatments. When contracting COVID-19, it was reported that cancer patients are 3.5-fold more likely to require mechanical ventilation or ICU admission. Therefore, they are more susceptible to death compared to COVID-19 patients that do not have cancer [23]. Published literature consistently reports that the critical predictor of cancer patients’ mortality when infected with SARS-CoV-2 are; gender, age, and comorbidity burden. In addition, some studies propose additional predictors. For example, one study reports that patients with hematologic malignancies are more susceptible to developing severe COVID-19 infection and death than those with solid tumors [24]. Another study examined COVID-19 clinical manifestation and death in relation to cancer treatment. The study reported that people undergoing cytotoxic chemotherapy are at a higher risk of clinical worsening and death. Whereas immunotherapy, hormone therapy, and targeted therapy did not influence clinical manifestation or death [25]. Others, however, reported that the type of anti-cancer therapy did not contribute as a mortality predictor [26, 27].

An increasing body of literature focused on understanding the effects of SARS-CoV-2 infection on chronic disease patients. Moreover, these studies attempted to determine the factors that affect the clinical prognosis of each high-risk group. The prevalence of CVDs, CKDs, and cancer and their impact on COVID-19 vary across different populations. Therefore, in this study, we collect and examine data from the Saudi population. We investigated COVID-19 characteristics, including symptoms and clinical manifestations, management, and outcomes among these vulnerable disease groups in the kingdom. The aim is to reduce the pathogen's effect and improve the prognosis for chronic disease patients infected with this virus and other similar pathogens.

## Study Design and Setting

Data for this retrospective, multicentre cohort study was collected from the electronic medical records of the National Guard Health Affairs (NGHA) hospitals, in Riyadh, Jeddah, Al-Ahsa, Dammam and Al-Madinah AlMunawarah, Saudi Arabia. Collection and analysis of data were done following approval from the Institutional Review Board (IRB) Committee-King Abdullah International Medical Research Center (KAIMRC)-NGHA (IRBC/2285/20). Patient confidentiality was protected by assigning an anonymous identification code; therefore, individual consent was not required. All patients’ related data was stored in a locked, password-protected file. The criteria for data collection were patients having laboratory-confirmed COVID-19 diagnosis upon a nasopharyngeal swab specimen (between March 2020 to January 2021), admitted to the ICU with one of the following conditions: CVD, CKD, or a type of cancer. Diagnosis of COVID-19 infection was based on the guidelines set by the Saudi Center for Disease Prevention and Control (SCDC). Those who tested negative for COVID-19, patients under 18 years, and patients without an outcome (discharged or expired) were excluded. Demographic information (hospital name, gender, and age), symptoms, vital signs, clinical and laboratory data, medication lists, and outcomes were extracted from the electronic medical records and analysed. Laboratory reference ranges of all parameters were based on the cut-offs used in the central laboratory of King Abdul-Aziz Medical City (KAMC), NGHA, Riyadh, Saudi Arabia.

## Data Analysis

Data were analysed using the statistical program SAS (version 9.4). Analysed data were presented as a frequency with a percentage (%) for categorical variables and mean ± standard deviation (SD) for continuous variables. Fisher’s Exact test was done for the association between categorical variables, and Wilcoxon Two sample test for continuous variables. All statistical tests were considered significant at *P* ≤ 0.05.

## Results

### Patients’ Characteristics

**Fig. 1:**
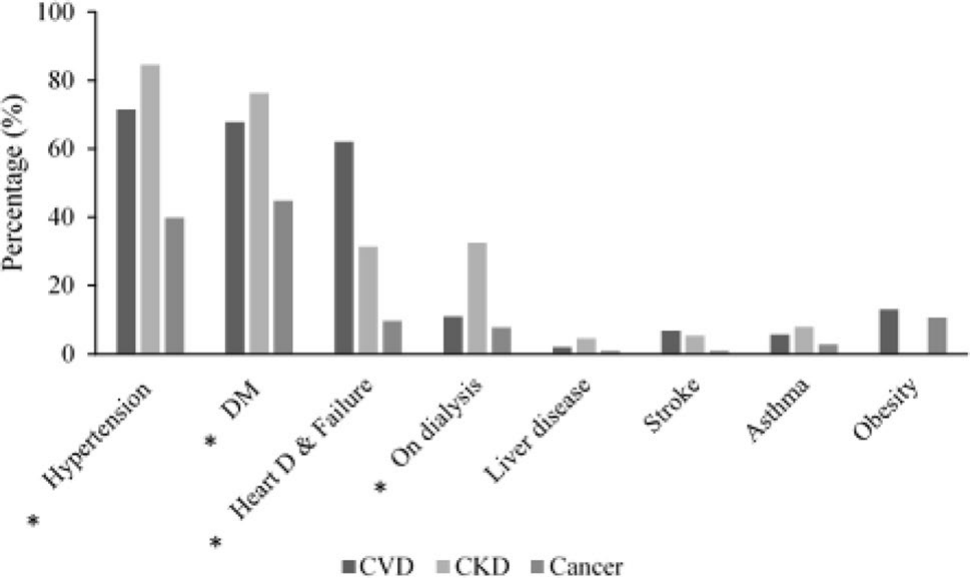
Other comorbidities of COVID-19-positive patients included in this study. The most common are hypertension, diabetes mellitus (DM), and heart disease/failure. Numbers indicate the total percentage of cases diagnosed with each comorbidity. *denotes significant value at *p* ≤ 0.05

A total of 535 patients were included in this study. Of which, 192 were CVD patients (M; 122, F; 70), 240 were CKD patients (M; 142, F; 98) and 103 were cancer patients (M; 38, F; 65). The mean age for each group was 65.2 ± 15.63 years for CVD patients, 68.08 ± 14.48 years for CKD patients, and 62.03 ± 13.44 years for cancer patients. Other common comorbidities in the selected samples are shown in Fig. 1. At the time of data collection, 433 patients were discharged from the ICU, and 102 patients were declared dead.

### Symptoms on Admission

On admission, most patients from the three groups examined in this study; CVD, CKD, and cancer patients, had a cough (55.73, 50.42, and 50.5%, respectively), shortness of breath (SOB) (59.38, 43.1, and 43.7%, respectively), and fever (41.15, 48.75, and 28.2%, respectively). Other COVID-19-related symptoms such as vomiting, diarrhea, nausea, headache, sore throat, chest pain, and myalgia were reported less frequently. Amongst the three groups, COVID-19 cases with CVD had a significantly higher prevalence of chest pain and SOB compared with COVID-19 cases that have CKD or cancer (*p*-value 0.0004 and 0.0017, respectively). While COVID-19 cases that have CKD had a significantly higher fever compared with COVID-19 cases that have CVD or cancer (*p*-value, 0.0016). Finally, COVID-19 cases with cancer reported a significantly higher myalgia rate compared with the other two groups (*p*-value < 0.001). There were no significant differences in the reporting of the other COVID-19-related symptoms between the three groups; see Table 1 for details.

**Table 1:** Common COVID-19-related symptoms as presented by CVD, CKD, and Cancer patients with COVID-19 upon ICU Admission

	CVD	CKD	Cancer	*P*-value
	Frequency (%)	Frequency (%)	Frequency (%)	
Overall	192 (100%)	240 (100%)	103 (100%)	
Cough	107 (55.73)	121 (50.42)	52 (50.5)	0.0643
SOB	114 (59.38)*	103 (43.1)	45 (43.7)	0.0017
Fever	79 (41.15)	117 (48.75)*	29 (28.2)	0.0016
Vomiting	24 (12.5)	47 (19.58)	16 (15.5)	0.1370
Diarrhea	19 (9.9)	24 (10)	11 (10.7)	0.9638
Nausea	20 (10.42)	23 (9.58)	11 (10.7)	0.9461
Headache	14 (7.29)	20 (8.33)	8 (7.77)	0.9553
Sore throat	17 (8.85)	15 (6.25)	5 (4.85)	0.3929
Chest Pain	36 (18.75)*	16 (6.67)	9 (8.74)	0.0004
Myalgia	42 (21.88)	4 (1.67)	32 (31.1)*	< 0.0001

### Clinical Characteristics

Table 2 displays the clinical laboratory investigations for the ICU- admitted CVD, CKD, and cancer patients that tested positive for COVID-19. Patients had readings outside the normal range for D-dimer, LDH in all three groups. For the renal profile, creatinine was elevated in CVD and CKD patients only. Interestingly, inflammatory markers, such as ferritin and C-reactive protein (CRP) showed a major elevation from the normal range among all three groups. While, troponin, was above the normal range in CVD patients, lower than the normal range in cancer patients, and remained within the normal range in CKD patients.

**Table 2:** laboratory findings of CVD, CKD, and Cancer- COVID-19 Patients upon ICU Admission

Lab findings	Normal range	CVD *N* = 192	CKD *N* = 240	Cancer *N* = 103	*P* value
Blood count					
Hemoglobin (gm/L)	M:140–180 gm/L, F:120–160 gm/L	119 (59)*	108 (45)	113 (25)	0.0033
WBC (X10_9/L)	4.0–11.0 X10_9/L	7 (5.68)*	6.34 (3.71)	6.29 (4.74)	0.0214
Platelets (X10_9/L)	140–450 X10_9/L	231 (124)	205 (115)*	243 (156)	0.0014
Lymphocytes (X10_9/L)	1.00–5.0 X10_9/L	1.27 (1.06)	1.24 (0.75)	1.01 (0.96)*	0.0099
D-Dimer (mg/L)	0.2–0.7 mg/L	0.98 (1.48)	1.05 (1.6)	1.12 (1.28)	0.6573
Albumin (g/L)	35–50 g/L	35 (7)	36 (6)	34 (8)	0.4914
Bilirubin (µmol/L)	3–22 µmol/L	9.8 (6.3)	7.3 (4.4)*	9.2 (6.8)	0.0001
LDH (U/L)	84–246 U/L	345.5 (226)	314.5 (176)	291.5 (152)	0.0808
AST (U/L)	15–37 U/L	31 (28)*	28.5 (23)	27 (21)	0.0373
ALT (U/L)	20–65 U/L	23 (23)*	20 (19)	21 (17)	0.0376
Renal profile					
Creatinine (µmol/L)	49–90 µmol/L	102 (93)	255 (399)*	68.5 (38)	< 0.0001
Inflammatory markers					
Ferritin (µg/L)	13–150 µg/L	394.55 (601.6)	518.97 (986.31)*	395.5 (560.6)	0.0118
CRP (mg/L)	< 10 mg/L	51.85 (107)	65.9 (95)	83 (99.4)	0.0861
Troponin (pg/ml)	24–30 pg/ml	32.65 (151.9)	24.6 (46.8)	11.12 (19.46)*	< 0.0001

*WBC* white blood count, *LDH* lactate dehydrogenase, *AST* aspartate aminotransferase, *ALT* alanine transferase, *CRP* C-reactive protein, Highlighted numbers are values outside the normal range; Significance represents differences between the 3 groups, NS not significant; significant at *p* ≤ 0.05

*denotes non-normal data and presented as Median (IQR Quartile Range)

As the main aim of this study is to examine the differences between the three chronic diseases, the clinical characteristics between these groups were compared. Data analysis revealed that CVD patients with COVID-19 had significantly different hemoglobin, WBC, AST, and ALT readings. CKD patients had significantly different platelets, bilirubin, creatinine, and ferritin readings. Whereas cancer patients had statistically different readings in lymphocytes and troponin levels compared with the other two patient groups.

### Management

At the ICU, most patients included in this study were given two or fewer types of medications. However, few of them were given more (Table 3). We observed that patients with other comorbidities are more likely to receive more than two drugs. For instance, CVD, CKD, and cancer patients received more than two medications when they are diabetic or hypertensive.

**Table 3:** Distribution of patients who received 0–2 vs. ≥ 3 medications in each group

	CVD (*n* = 192)	CKD (*n* = 240)	Cancer (*n* = 103)	*P*-value
0–2 medications	179 /192 (93.23%)	180/240 (75%)	100/103 (97.09%)	< 0.0001
3 or more medications	13/192 (6.77%)	60/240 (25%)	3/103 (2.91%)	

**Fig. 2:**
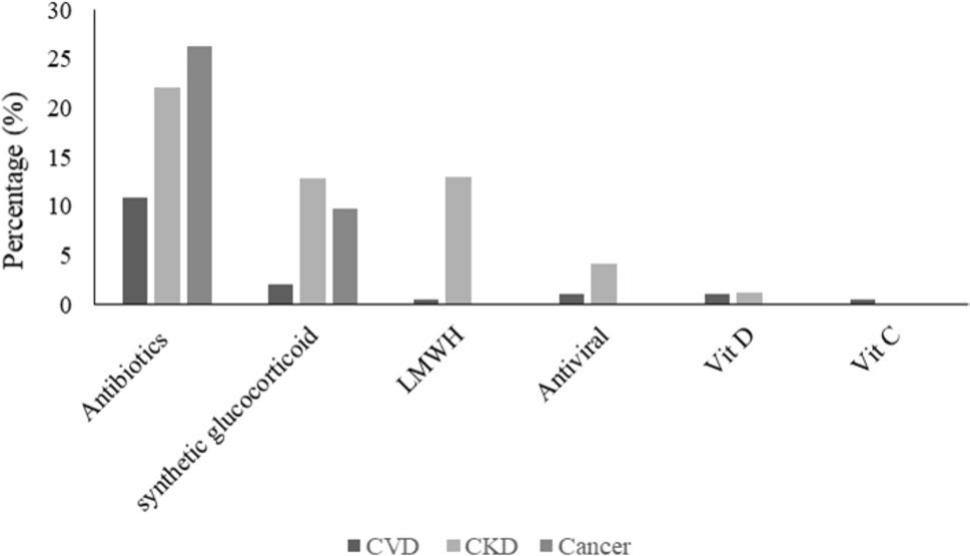
Management of CVD, CKD, and cancer-ICU admitted-COVID-19 patients. The bar graph shows the percentage of patients who received X medication. *denotes significant value at p ≤ 0.05

The type of medicine given to the patients during the ICU stay is shown in Fig. 2. The three most commonly administered drugs are antibiotics, synthetic glucocorticoids, and low molecular weight heparin (LMWH). Other options include antiviral, vitamin C and D, methylprednisolone, and Favipiravir. Antibiotics are the most commonly prescribed among the numerous drugs used during the treatment of COVID-19-patients. Doxycycline and ceftriaxone are two examples of antibiotics given to COVID-19-positive patients in the ICU. Our results showed that 83.87% of CVD, 83.75% of CKD and 99.03% of cancer patients were on antibiotics during their stay at ICU (*p*-value < 0.0001). Synthetic glucocorticoids such as Dexamethasone were the second option used to treat COVID-19 patients. The percentage of CVD, CKD, and cancer patients who received Dexamethasone during ICU stay was 20.97%, 34.17%, and 17.5%, respectively (*p*-value, 0.0007). COVID-19 patients are at high risk of developing coagulopathies, which increases upon admission to the ICU. Therefore, the use of anticoagulants such as heparin is highly recommended. Most patients who received LMWH were CKD patients (37.5%), followed by CVD patients (3.23%). While none of the cancer patients received LMWH. Here in our study, antiviral drugs were given to a small proportion of admitted patients within the three groups. That is 3.76%, 7.50%, and 2.91% of CVD, CKD, and cancer patients, respectively. Moreover, vitamin C and D were other options for managing COVID-19 infection. Interestingly, in our study, we have noticed that vitamin D was given more than vitamin C.

### Length of ICU Stay and Outcomes

The ICU length of stay (LoS) varied between patients with CVD and cancer compared with patients with CKD. The LoS was 11.01 ± 9.98, 13.93 ± 15.87, and 11.12 ± 8.79 days for CVD, CKD, and cancer patients, respectively. Data analysis revealed that longer ICU LoS correlates with poorer outcomes. Discharged patients had a shorter stay at the ICU compared with patients who died in the ICU. For CVD and cancer-COVID-19-positive patients, ICU LoS in discharged vs. dead patients was not changed significantly. However, ICU LoS in CKD COVID-19-positive patients increased significantly in expired compared to discharge patients. The average time from ICU admission to discharge was 12 ± 15.46 days. However, it significantly increased to 20.72 ± 15.58 days for CKD patients from ICU admission to death (*P* < 0.0001).

The mortality rate increased among patients with comorbidities. Here in our study, the data showed that 91.30% of expired CVD patients were hypertensive. Moreover, since CKD patients had a higher mean age, age was another factor that increased the risk of death in the ICU. Our results showed that older CKD patients were at higher risk of dying from COVID-19 infection in the ICU. The average age of discharged CKD patients was 65.42 ± 14.76 years. However, it increased significantly in dead patients to 75.92 ± 10.24 years (*P* < 0.0001).

## Discussion

In this retrospective study, we investigated the COVID-19 infection characteristics in CVD, CKD, and cancer patients. In the examined population, the number of males was slightly higher than females (56.4% vs. 43.6%), similar to another published national study [28]. The mean age for each group was 65.2 ± 15.63 years for CVD, 68.08 ± 14.48 years for CKD, and 62.03 ± 13.44 years for cancer patients. This is in agreement with published literature from China [29] and the United States [30], while other studies of the Saudi population showed a lower median age of 36 years [31]. The existence of comorbidities is a significant risk factor for COVID-19 severity. Like many previous reports [31, 32], in our study, DM, hypertension and heart disease/failure were the most common comorbidities seen in patients. The overall mortality rate among patients included in this study was only 19% by January 2021, while the rest were discharged from the hospital due to effective control and treatment of COVID-19 infection.

Most patients were symptomatic and presented with standard COVID-19 symptoms, including cough, SoB, and fever as the most common symptoms among patients, supporting the observations of previous studies. COVID-19 cases that have CVD had a significantly higher prevalence of chest pain and SoB. This finding is probably because chest pain and SoB are frequent symptoms among CVD patients even without viral infection [33]. The prevalence of fever was also more in COVID-19 cases that have CKD compared with COVID-19 cases that have CVD or cancer. This observation could be because most CKD patients in this study were on dialysis. Fever is common among patients on dialysis and is usually attributed to respiratory and digestive infections [34]. While COVID-19 cases with cancer reported a significantly higher myalgia rate. This is not surprising since pain is common in all cancer types and affects 37%-64% of cancer patients. The pain is due to tissue damage produced by tumour cells and toxic side effects of chemotherapy and radiotherapy treatments on the neural structures [35].

To identify biochemical markers that are significant for the progression of the infection in each group, laboratory tests results were examined for all cases included in our study. It has been shown previously that the level of variation in the laboratory results of COVID-19 patients is different among many studies [36], Here, it was observed that the levels of D-dimer, LDH, renal profile and Inflammatory markers, such as ferritin, CRP, and troponin were outside the normal range. This is not surprising given the fact that the mean age of COVID-19 patients in this study was above 60 years old, with chronic comorbidities and a weak immune system to fight the viral infection, which led to an abnormal variation of some of the laboratory results. Additionally, high levels of D-dimer, LDH, ferritin and CPR are reported in most published studies and considered as indicators of COVID-19 progression and severity [32, 37]. Qin et al. reported a major link between high levels of CRP and the severity of the COVID-19 infection [30]. Moreover, elevated D-dimer, LDH, and CRP levels were associated with adverse side effects of COVID-19 including high risk of acute respiratory distress syndrome (ARDS), ICU admission, and mortality [38].

As for management, antibiotics were prescribed to most patients during their stay at the ICU. Especially cancer patients since they are immunocompromised and highly susceptible to bacterial co-infection [39]. Some Antibiotics, such as Doxycycline and Ceftriaxone, are safe and effective against viral pathogens. They are known to regulate and block pathways essential for viral replication [40]. Doxycycline, in particular, has been proposed as a treatment for COVID-19 [41], and was commonly used in this study. Still, data from the US and the UK suggested that there has been an overuse of Doxycycline for treating COVID-19. Also, in our study, most patients in the ICU were treated with a combination of antibiotics. This could cause antimicrobial resistance and therefore cause global public harm. Synthetic glucocorticoids were the second option used to treat COVID-19 patients in our study. Dexamethasone is a glucocorticoid that can limit the effect of cytokines. Therefore, giving COVID-19 patients Dexamethasone can regulate lung inflammation and reduce acute lung diseases' progression. However, it will also inhibit the functions of T and B cells and prevent them from making antibodies which causes an increased viral load that will continue after the patient has survived [39]. Therefore, it is only recommended for the short term in severe cases. Although antiviral drugs, such as Remedisivir, have been used only on a small proportion of patients in our study, they may have some benefits. The usage restriction of those drugs is expected due to their potential toxicity, limited availability, and high cost [39].

Finally, we investigated the correlation between LoS at the ICU and patients’ outcomes. Discharged patients had a shorter stay at the ICU compared to patients who expired. This was more prominent in CKD COVID-19-positive patients, where the LoS at the ICU increased significantly in expired compared with discharged patients. It has been reported in previous studies that CKD patients hospitalized with infections have resulted in increased ICU admissions and LoS in hospitals and subsequently higher mortality rates compared with those without CKD [42]. Some factors associated with the CKD patient group observed in this study may explain the link between CKD and poor clinical outcomes. This includes having a higher mean age and more comorbidities compared with the other two groups. In addition, most CKD patients in this study are under haemodialysis, which may lead to poorer response to treatment, immune dysfunction with higher susceptibility to infections, and more prevalence of dehydration which may lead to acute kidney injury [42]. Moreover, high susceptibility to infections may lead to more use of antibiotics. This could increase the risk of antibiotic-resistant pathogens in those patients and subsequently leads to a longer LoS in the ICU and thus high mortality rate [43].

## Conclusion

This retrospective study is the first study done in Saudi Arabia that comprehensively investigates the clinical characteristics, severity, and outcome of ICU admitted-COVID-19 patients with three different chronic diseases. It provides robust and valuable findings on how these different chronic diseases directly affect the severity of COVID-19 infection. Our data revealed significantly different disease patterns in symptoms, laboratory findings, required treatments and outcomes between these three groups of patients. Highlighting the major death indicators among COVID-19 patients with CVD, CKD and cancer, will provide physicians with comprehensive guidance. This is crucial to predict the infection's severity and complications among chronic disease patients. It can also aid in prioritizing ICU admission. Moreover, data from this study will add to the growing body of literature on COVID-19 manifestation in Saudi population, which will enable the implementation of accurate control strategies in the future.

## Study Limitations

We acknowledge several limitations in this study. First, the study was retrospective in design, limited to a single healthcare system, and included only 535 confirmed cases, which is considered a relatively small sample size. Moreover, it was carried out over a short time, and no data were collected after the patients were discharged. In the future, enormous prospective, multi-center studies are required over extended periods and larger sample sizes. Second, we could not eliminate recall bias as findings constrain the accuracy of data collection and recording. Therefore, the correctness of the onset of symptoms, medical history, comorbidities, and used medications was based on the patient’s account and hospital records. Third, several major parameters such as BMI, DM type, cancer type and stage, medications for existing comorbidities, and some clinical characteristics such as CT scan of the chest were not included in our data. This may have affected the findings and added clinical value to the study. Fourth, the diversity and heterogeneity among CVD, CKD, and cancer patients including their age and comorbidities reduce the uniformity of the study population. Finally, given the retrospective nature of this study, data were collected before the COVID-19 vaccination. Therefore, a similar future study investigating the effectiveness of the COVID-19 vaccine among Saudi patients with chronic diseases is necessary.

## Data Availability

Data is available upon request.

## Abbreviations

COVID-19: Coronavirus disease
WHO: World Health Oragnization
CFR: Case fatality rate
CVD: Cardiovascular diseases
ACE2: Angiotensin converting enzyme 2
ICU: Intensive care unit
CKD: Chronic kidney diseases
AKI: Acute kidney injury
DM: Diabetes mellitus
SoB: Shortness of breath
LMWH: Low molecular weight heparin
LoS: Length of stay
